# Cost-effectiveness analysis of proximal femoral nail versus bipolar hemiarthroplasty for femoral neck fracture

**DOI:** 10.1186/s13018-024-04941-3

**Published:** 2024-08-27

**Authors:** Gamze Kutlu, Yasemin Akbulut

**Affiliations:** 1 Department of Health Management, Faculty of Economics and Administrative Sciences, Yozgat Bozok University, Yozgat, Turkey; 2 Department of Health Management, Faculty of Health Sciences, Ankara University, Ankara, Turkey

**Keywords:** Cost-effectiveness, Health policy, Health management, Quality of life, Reimbursement

## Abstract

**Background:**

Hip fractures are a serious public health problem with high rates of morbidity, mortality, disability and care costs. The aim of the research was to perform cost effectiveness analysis of hip fracture treatments using proximal femoral nail and bipolar hemiarthroplasty surgeries.

**Methods:**

The analysis was completed based on the perspectives of the paying institution and patient. A decision tree model was used to determine whether proximal femoral nail or bipolar arthroplasty was most cost effective for the management of a femoral neck fracture in this patient population.

**Results:**

The findings from the decision tree model suggested that ICERs for BHP were TRY 43,164.53 TL/QALY based on reimbursement and TRY 3,977.35 TL/QALY based on patient expenditures. Compared to the calculated threshold value of TRY 60.575 TL, we concluded BHP to be a cost-effective option. Moreover, all parameter changes yielded stable results on the one-way sensitivity analysis. When it comes to the probabilistic sensitivity analysis, BHP with specified threshold value was found to be cost-effective in all the comparisons. Currently available data the use of bipolar hemiarthroplasty as the more cost- effective treatment strategy in this specific population.

**Conclusion:**

Overall, our findings showed HA as a cost-effective surgical technique at the calculated threshold in a population over 60 years of age. The impacts of HA on patients’ quality of life and costs are remarkable.

## Introduction

Health technology assessment (HTA) assists national health systems in deciding how to allocate different health technologies or alternatives with limited resources [1]. No country, including developed countries, has adequate resources to provide all health requirements for the population [2]. Resources must be effectively used to ensure more progress in the field of health, to overcome new difficulties and to resolve inequality. To ensure this, there is a need for information about which interventions really work, how much they cost, and about their application and presentation [3]. In this context, costs and outcomes should be defined to assess the added value of new technologies appropriately. It provides an important tool to assess the clinical and cost effectiveness of health service interventions [1, 4].

Since the second half of the twentieth century, the incidence of hip fracture observed in the population aged over 65 years has become a notable problem for health systems. Linked to the extension of life expectancy at birth, the proportion of the elderly population has increased in countries. It has been reported that the pace of population ageing is much faster than in the past and that by 2020 the number of people aged 60 and over will exceed the number of children under 5. It is also projected that between 2015 and 2050, the proportion of the world population aged 60 + will almost double, from 12 per cent to 22 per cent [5]. In this context, the number of hip fractures in Türkiye and the world is expected to increase with the increases in the aging population and life expectancy. Problems emerging with aging like reduced physical capacity, weakened reflexes, and vision/hearing loss in the elderly weaken their protection and escape functions from environmental hazards. The reduction in bone density linked to age increases the risk of fractures [6]. Additionally, muscle and skeletal system diseases comprise nearly 30% of the global disease burden and form an increasingly heavy economic burden on the health system, especially with an aging population [7]. In 1990, 1.6 million hip fractures are known to have occurred globally. This number is predicted to be above 6 million in 2050 [8]. Türkiye is one of the countries with lowest incidence of hip fracture in Europe, though a clear increase was observed in recent years. The FRACTURK study reported that predicted hip fracture numbers were 24,000 in 2009 in Türkiye and will be 64,000 in 2035 [9]. This scenario is associated with a greater burden in terms of both loss of health, and social and health care costs. In Europe, hip fracture cases are stated to cause more disability-related life years than many common types of cancer. At the same time, high-cost procedures are performed for surgical care, medical care and rehabilitation of hip fractures [10]. As a result, the choice of which surgical method to use for femur neck fractures in the elderly is a frequently debated and researched topic. Accordingly, it is important to identify the true effects of alternative treatment methods on individual and public health to be able to control health spending.

The aim of the study is to analyze the cost-effectiveness of hip fracture treatments using proximal femoral nail (PFN) and bipolar hemiarthroplasty (BHA) operations. There are some strengths that distinguish our study from other paper. Firstly, there are studies in the literature on the cost-effectiveness of different alternatives used for hip fracture treatment. In this context, there are cost-effectiveness analyses of partial hip replacement and total hip replacement [11, 12], standard implant and advanced implant [13], cemented and uncemented total hip replacement [14, 15] and timely treatment and late treatment [16, 17]. There are also cost-effectiveness studies comparing hemiarthroplasty and internal fixation methods [18–21] however, it has been stated that these studies are missing for evidence-based literature due to heterogeneity [22]. In particular, the lack of studies on the cost-effectiveness of PFN and BHA methods and the need to compare homogeneous groups reveal the importance of this study. Secondly, the fact that the research is conducted from 2 different perspectives constitutes another strong aspect. Finally, the budget impact analysis complemented the cost-effectiveness analysis by revealing its impact on the budget. Our research will provide evidence-based information to payers, service providers and patients.

## Materials and methods

We structured this part of the study according to CHEERS checklist [23].

### Comparators

While there exist various treatment options for patients with femoral neck fractures, arthroplasty and internal fixation methods remain the primary approaches. Typically, internal fixation is recommended for younger patients with robust bone quality and good overall health. However, there continues to be a debate surrounding the most suitable treatment choice for older patients, with no consensus established as of yet [19, 24]. In this study, we compared the treatment options of bipolar hip arthroplasty with the proximal femoral nail internal fixation method.

### Target population

The research was completed in the Orthopedic Clinic of XXBLINDEDXX. This cross-sectional study was conducted with patients aged 60 and over who would undergo PFN or BHA surgery as treatment for hip fracture between October 2019 and December 2020. Data collection was suspended for approximately 3 months due to the COVID-19 outbreak within the planned date range. The research group consisted of a total of 145 patients, 88 of whom had PFN and 57 of whom had BHA surgery.

### Measurement of effectiveness

In the study, we used the EQ-5D-5 L health questionnaire to measure health-related quality of life. We obtained permission from EuroQoL to use the questionnaire and they provided the Turkish version. In the second part of the questionnaire, we asked patients to give a score from 0: worst to 100: best, representing their health status on that day. We administered the questionnaires to the patients in two stages. In the first stage, we obtained information about the patients diagnosed with hip fracture from the surgery list in the orthopedic clinics and the surgery decision. In this context, we received information about the room numbers and the general condition of the patients after the interviews with the doctors every morning. We contacted the patients, informed the patient or caregiver about the study, obtained their consent, and administered the EQ-5D-5 L preoperatively to the patients who volunteered to participate in the study. In the second stage, we reapplied the same questionnaire to the operated patients at the end of 3 months in line with expert opinion and literature research [25]. Since we received cell phone numbers from the patients and their relatives in the first interview, we then obtained the information by making phone calls with the patients. In this way, we determined the quality of life related to health after surgery.

### Estimating resources and costs

In the research, cost data encompassed direct medical costs and non-medical costs. Accordingly, two data sources were used for cost data. We collected data on patients’ out-of-pocket expenditures through a “direct cost questionnaire”. We calculated patients’ out-of-pocket expenditures based on total costs shown on invoices provided by the patients or based on declaration. The declared amount was based on transportation, food and accommodation expenses of patients and their relatives. We also calculated transportation costs by personal vehicles by multiplying the unit cost of gasoline by the distance traveled. In this way, mean non-medical costs were directly determined from the perspective of the patient.

For calculation of direct medical costs from the payer perspective for hip surgery in the research, the “invoice form” for each patient was used. In Türkiye, both treatment methods are covered by the guarantee packet of the Social Security Institution (SSI). Data were obtained using a computer allocated to the researcher by scanning and examining the days (total of 26 days) determined according to the work schedule of the cost analysis and billing units.

Within the scope of the research, we calculated the amount and frequency of medications used, imaging methods, and outpatient clinic visits in order to determine the cost of a patient who did not experience any complications after discharge. We obtained from the hospital information system the number of times patients came for check-ups. The unit cost of drugs used after discharge was calculated using the costs in the current price list of the Turkish Medicines and Medical Devices Agency (TMMDA). When calculating the cost of drugs used after discharge, the public discount rate was determined and the value of one (1) Euro was taken as 4.5786 Turkish Lira (TL) [26]. In addition, revisions that may develop outside the normal clinical course and patient mortality during treatment were determined. In this context, the costs of patients undergoing revision were obtained from the hospital information system.

### Study perspective

A health economy assessment may be performed from one or more perspectives like social perspective, public health perspective, health system perspective, payer perspective, institutional perspective and/or patient perspective [27]. In this research, the alternative methods used for hip fracture surgery were investigated from payer (SSI) and patient perspectives.

### Time horizon and discount rate

When costs and effects are assessed, it was stated that reductions should not be performed in studies with a time interval of less than one year (pediatric flu vaccination, conjugate pneumococci, etc.) [28]. In this context, a reduction rate was not used as the study assessed the costs and effects of hip fracture surgery methods with a 1-year horizon.

### Research design and model

**Fig. 1:**
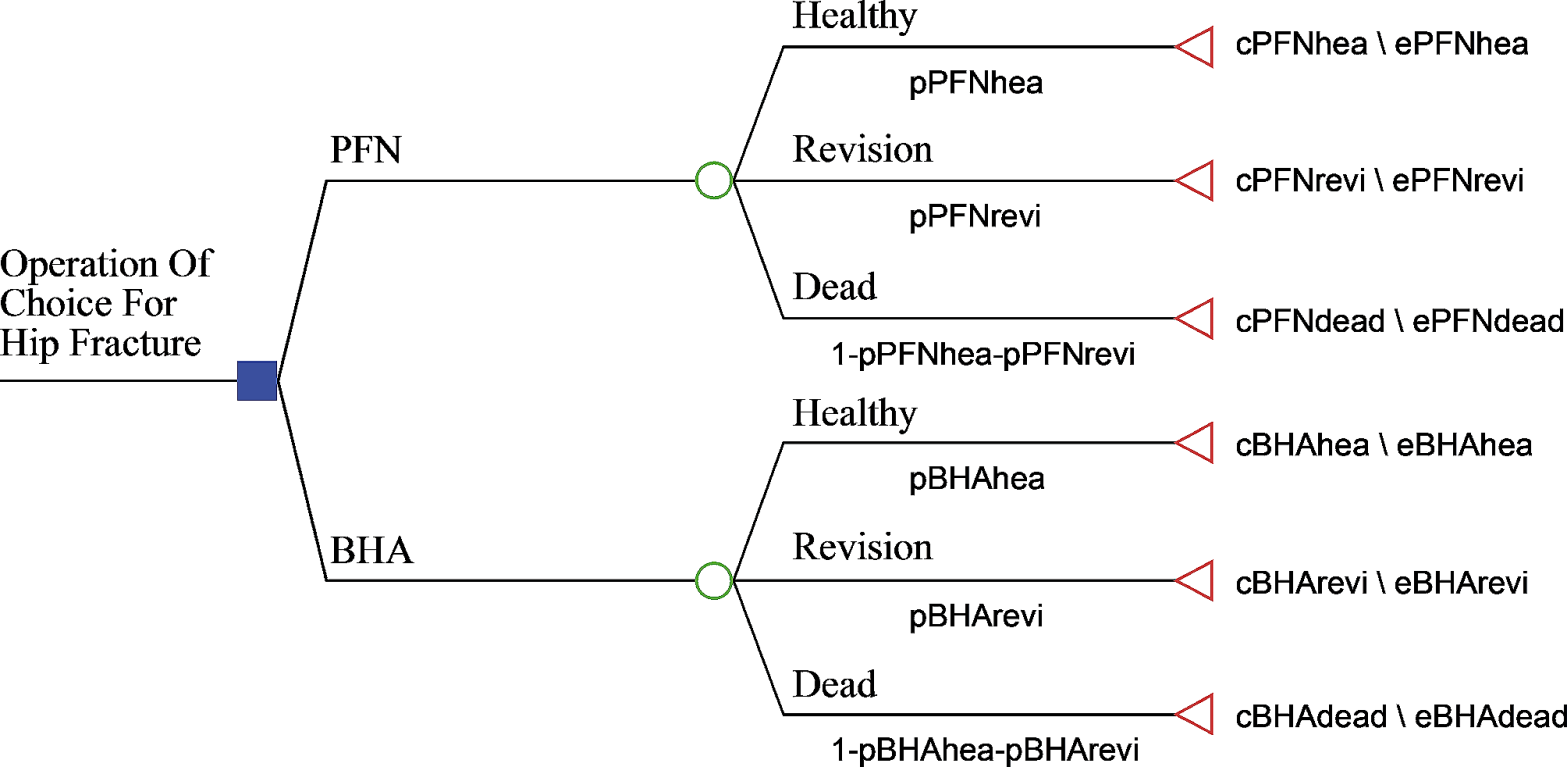
Research model

In design of the research, the decision tree model was used to be able to compare cost effectiveness for two treatment approaches in patients with hip fracture and be able to make the best decision (Fig. 1). The situations defined in the decision tree model were determined with information obtained from the literature and expert opinion [12, 20, 29]. When creating the decision tree model for cost effectiveness analysis, the “TreeAge Pro-2021 (Healthcare Version)” program was used.

### Statistical analyses

Kolmogorov-Smirnov and Shapiro Wilk tests were used to ensure that the data conformed to normal distribution (*p* < 0.05). The effectiveness and cost ratios of each alternative compared were reported with the ICER. In health economic evaluations, it is becoming common to calculate the net monetary benefit (NMB) for alternatives in addition to, and sometimes instead of, the ICER. In this context, NMB results were included in order to make a more transparent comparison of the alternatives. Net benefit calculations are best for combining cost, effectiveness, and willingness to pay into a single measurement. Following the cost effectiveness analysis, sensitivity analysis and budget impact analysis, which are complementary to the cost effectiveness analysis, were carried out. Tornado diagram was used in sensitivity analysis and acceptability curves were presented. Ethics committee approval and permission from the relevant institution were obtained to perform the research.

## Results

Socio-demographics of the patients in the study group and information about the operation are given in Table 1.

**Table 1:** Socio-demographic and clinical characteristics of the Research Group*Sd: standard deviation*,* F: female*,* M: male*, $\:\bar{X}$: *mean*

Variable	Proksimal femoral Nail (*n* = 88)	Bipolar Hemiarthroplasty (*n* = 57)	All (*n* = 145)	*p*
*Mean age (sd)*	78.92 (10.21)	76.98 (12,18)	78.15 (11.03)	
*Gender (F: M)*	52:36	42:15	94:51	
*History of hip fracture (n*,* %)*				
*Yes*			22 (15.2)	
*No*			123 (84.8)	
*Whether as a result of falling (n*,* %)*				
*Yes*			134 (92.4)	
*No*			11 (7.6)	
*Hospitalization Days (*$\:\bar{X}$, *sd)*	6.42 (4.808)	6.14 (3.823)	6.31 (4.430)	0.998
*ASA Score (*$\:\bar{X}$, *sd)*	2.71 (0.605)	2.70 (0.596)	2.71 (0.599)	0.998
*Operation Time. min (*$\:\bar{X}$, *sd)*	75.05 (20.249)	78.17 (18.758)	76.28 (19.670)	0.376
*Number of Comorbidities (n*,* %)*				
*None*	32 (36.36)	15 (26.32)	47 (32.42)	
*1*	21 (23.86)	11 (19.30)	32 (22.07)	
*2*	12 (13.64)	9 (15.79)	21 (14.48)	0.372
*3*	8 (9.09)	12 (21.05)	20 (13.79)	
*4*	9 (10.23)	7 (12.28)	16 (11.03)	
*5 +*	6 (6.82)	3 (5.26)	9 (6.21)	

### Cost-effectiveness analyses

After determining the QALY values, costs and probability values for the PFN and BHA methods, cost effectiveness analysis was completed with the decision tree model created in the TreeAge program. The probability values (p), costs (c) and effectiveness (e) scores are represented on the branches of the decision tree (Table 2). These calculations were performed from the perspective of the repayment institution in the 1st model (direct medical costs) and of the patient in the 2nd model (direct non-medical costs).

**Table 2:** Parameters used in the decision Tree

Decision Tree Branches	Probability values	Source
p1-PFN Good health	0.7727	Research data
p2-PFN Revision	0.0796	Research data
p3-PFN Death	1-(p1 + p2)	
p4-BHA Good health	0.7719	Research data
p5-BHA Revision	0.1053	Research data
p6-BHA Death	1-(p4 + p5)	Research data
**Decision Tree Branches**	**Cost (TL)**	
c1.1-PFN Good health	6.305.19	Research data
c1.2-PFN Revision	11.516.93	Research data
c1.3-PFN Death	7.927.01	Research data
c1.4-BHA Good health	7.396.00	Research data
c1.5-BHA Revision	9.742.86	Research data
c1.6-BHA Death	12.543.82	Research data
c2.1-PFN Good health	359.09	Research data
c2.2-PFN Revision	310.43	Research data
c2.3-PFN Death	305.38	Research data
c2.4-BHA Good health	548.39	Research data
c2.5-BHA Revision	350.33	Research data
c2.6-BHA Death	67.79	Research data
c3.1-PFN Good health	6.664.28	Research data
c3.2-PFN Revision	11.827.36	Research data
c3.3-PFN Death	8.232.40	Research data
c3.4-BHA Good health	7.944.39	Research data
c3.5-BHA Revision	10.093.19	Research data
c3.6-BHA Death	12.611.61	Research data
**Decision Tree Branches**	**Effectiveness (QALY)**	
e1-PFN Good health	0.627	EQ-5D-5 L
e2-PFN Revision	0.538	EQ-5D-5 L
e3-PFN Death	0	
e4-BHA Good health	0.654	EQ-5D-5 L
e5-BHA Revision	0.503	EQ-5D-5 L
e6-BHA Death	0	

#### Model 1 analysis results

**Fig. 2:**
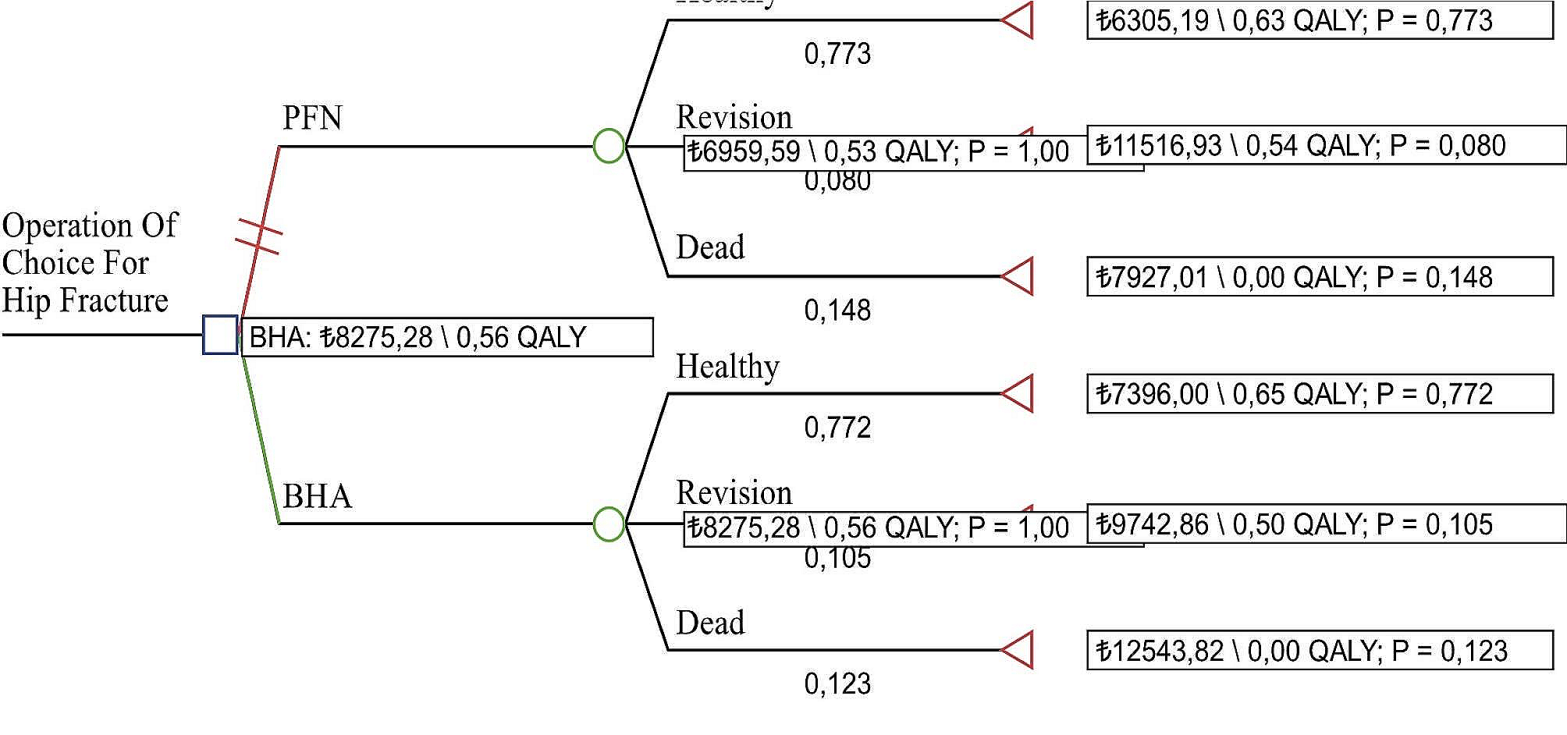
Model 1 decision tree

**Fig. 3:**
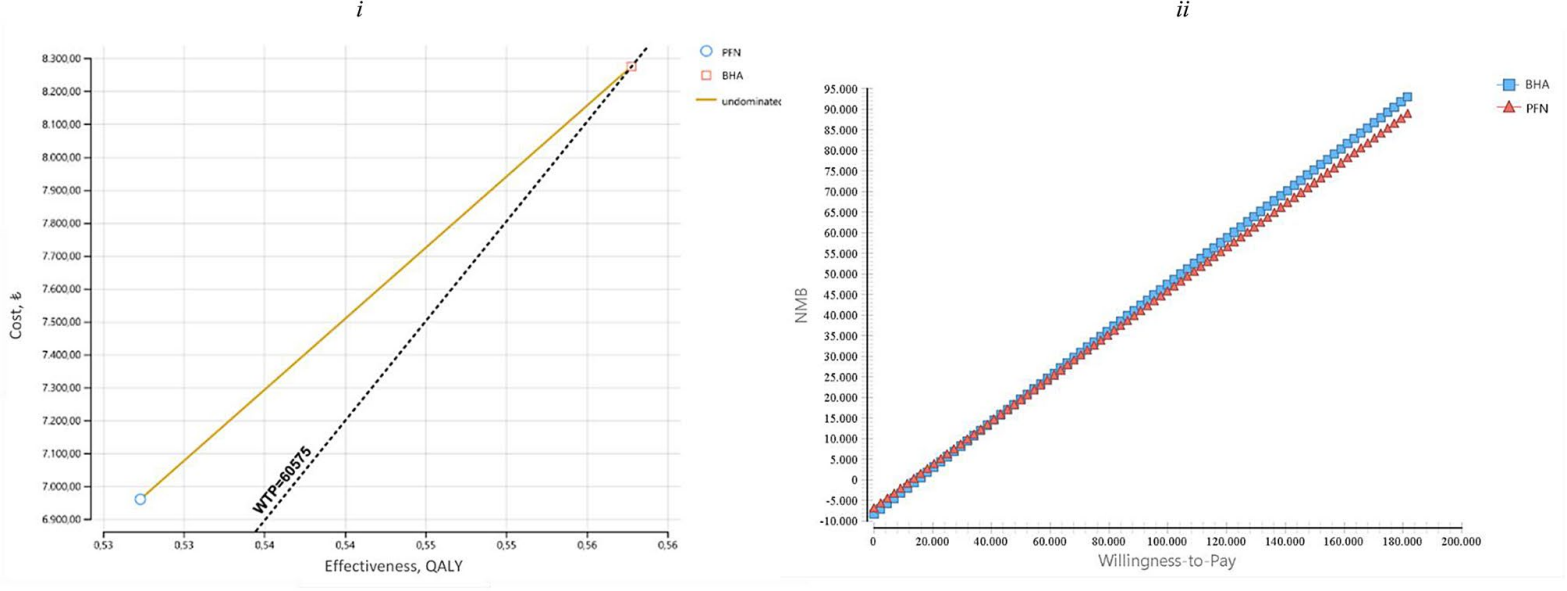
Model 1: cost effectiveness distribution and nmb

The BHA methods required 1315.69 TL additional costs compared to the PFN method and the additional cost effectiveness ratio for BHA was identified as 43,164.53 TL/QALY. In line with this, to gain additional QALY with the BHA method, it is necessary to spend 1315.69 TL (Table 3). When the decision tree model was examined, it was seen that the cost of BHA was higher than PFN and similarly its effectiveness was higher. In other words, the BHA method was found to be the optimal choice from the payer’s perspective (Fig. 2). ICER is in the 1st quartile of the cost-effectiveness plane. Accordingly, it should be noted that BHA is both more effective and costly and is one of the most frequently taken decisions (Fig. 3_i_). The NMB-WTP plot was used to observe how the optimal strategy changes when the cost-effectiveness threshold is changed. The net monetary benefit of PFN and BHA is quite close; However, for the threshold value of 40,854.38 TL and above, BHA was seen to have increased NBM (Fig. 3_ii_). In other words, it turns out that BHA provides the best bang for your buck.

**Table 3:** Cost effectiveness results relative to Direct Medical and non-medical costs

Operation method	Cost	Additional cost	Effectiveness (QALY)	Additional effectiveness	NMB	ICER
*1st model*						
PFN	6.959.59		0.53		19.405.80	
BHA	8.275.28	1.315.69	0.56	0.03	19.614.15	43.164.53
*2nd model*						
PFN	347.28		0.53		658.60	
BHA	468.52	121.23	0.56	0.03	839.95	3.977.35

#### Model 2 analysis results

**Fig. 4:**
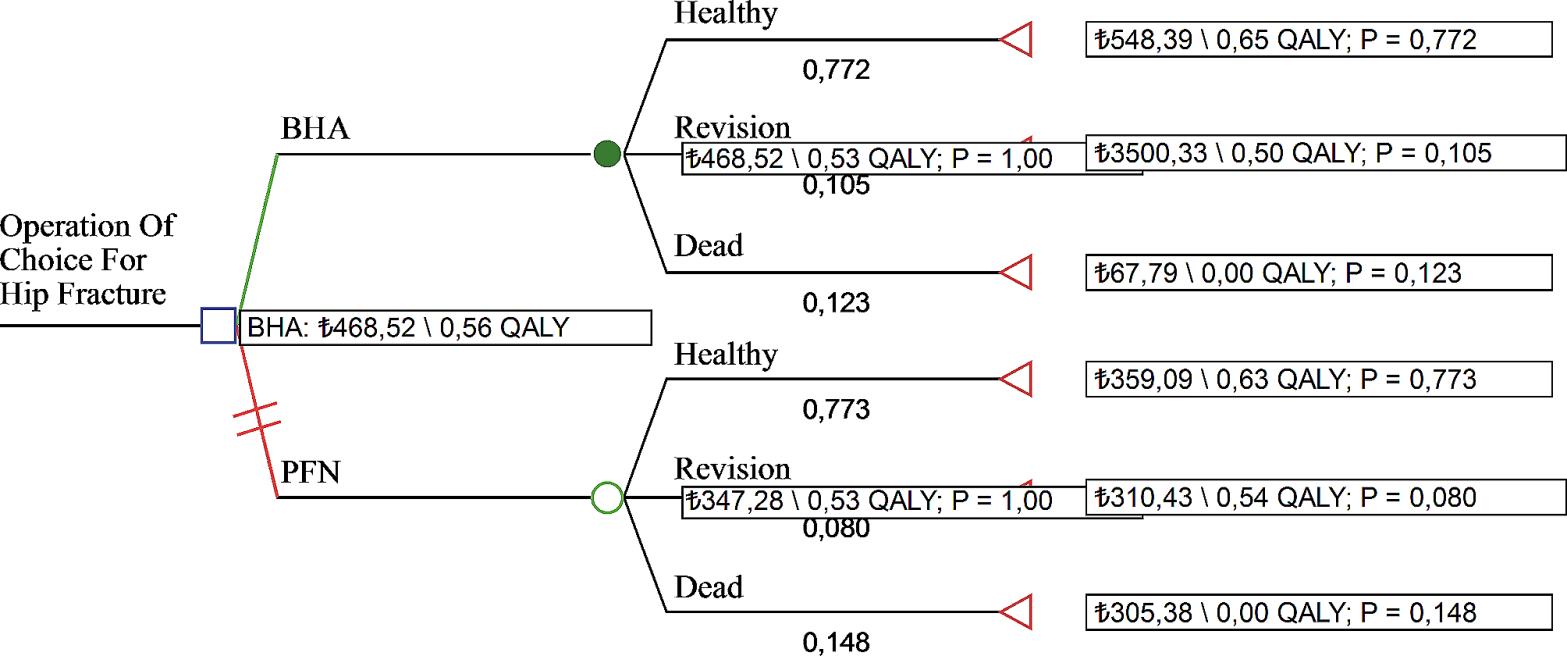
Model 2 decision tree

It was determined that BHA required an additional cost of 121.23 TL compared to PFN. When the effectiveness of the alternatives was compared, it was calculated that BHA had 0.03 more QALYs compared to PFN. In addition, the additional cost effectiveness ratio of BHA compared to PFN was determined as 3977.35 TL/QALY. In parallel with this, BHA needed to spend 121.23 TL to obtain additional QALY (Table 3). When the decision tree of Model 2 was analyzed, it was seen that both the cost (121.23 TL) and efficiency (0.56 QALY compared to 0.53 QALY) of BHA were higher (Fig. 3). In line with these results, the ICER value is located in the 1st quadrant of the cost-effectiveness plane. The fact that the ICER value is in the 1st quadrant of the cost-effectiveness plane shows that BHA is more costly and more effective than PFN. At this point, it is up to the decision maker to choose between the alternatives. Accordingly, if BHA treatment is expensive but health gains are achieved (higher effectiveness), the payer will decide whether it is worth paying for this treatment option. In this context, with an additional budget of 121.23 TL and 3977.35 TL/QALY, BHA can be selected if it is affordable, and if not, the cheaper PFN method can be selected. As a result, the willingness to pay threshold will guide decision makers. Accordingly, BHA turned out to be a cost-effective choice (Fig. 4i). The most cost-effective treatment is the one with the highest NMB value. Accordingly, it is seen that PFN and BHA have very close net monetary benefits. However, it was observed that the NBM value of BHA increased above the threshold value of 4538.12 TL. It was determined that the NMB of BHA increased as the threshold value increased (Figs. 4ii, 5). In conclusion, the results of the cost-effectiveness analysis from the patient perspective revealed that spending money on BHA provided better value.

**Fig. 5:**
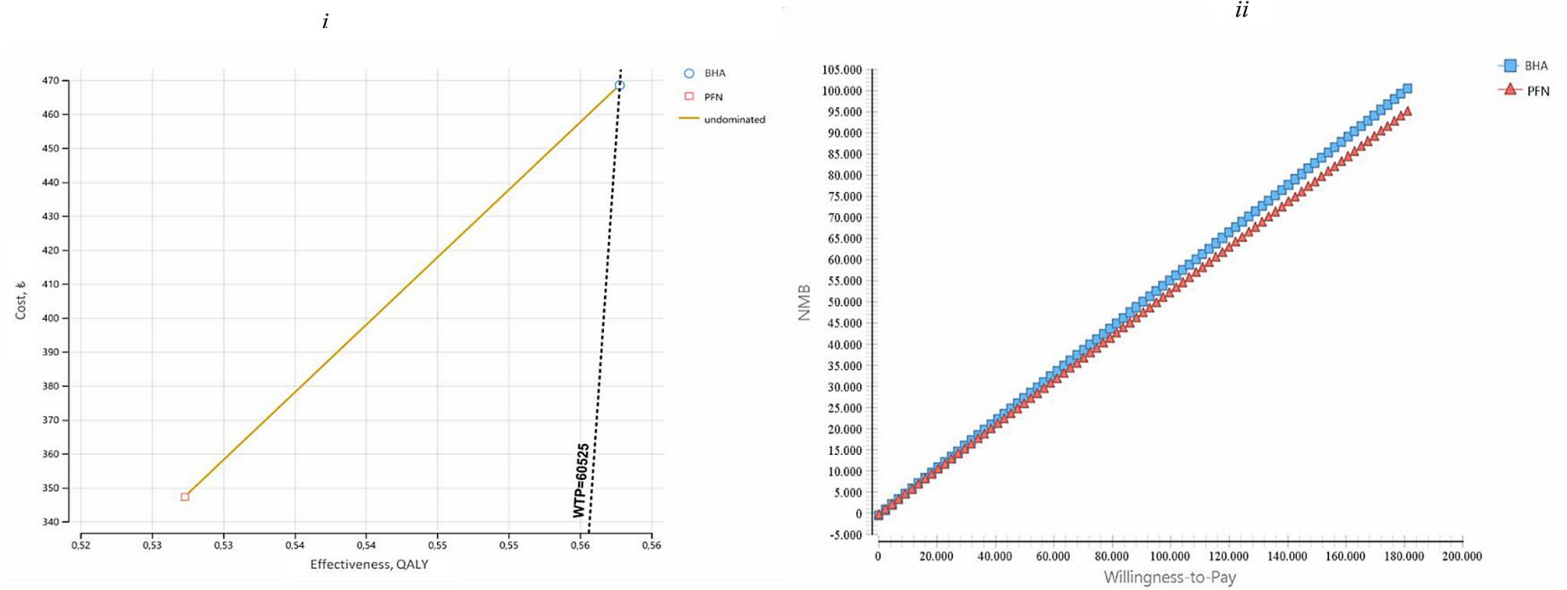
Model 2: cost effectiveness distribution and NMB

### Sensitivity analyses

#### Model 1

**Fig. 6:**
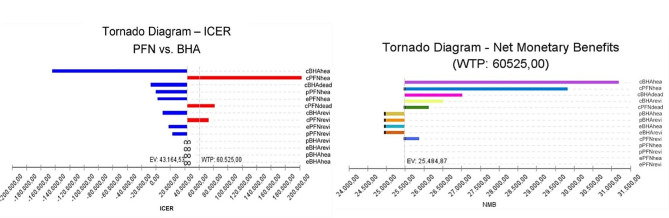
Model 1-tornado diagram

We find that the change in BHA and PFN costs has the largest impact on ICER (Fig. 6_i_). The highest line is the cBHAlive variable below the threshold value. The blue part of the line represents the part where the uncertainty range is low. The second highest line is the cPFNlive variable with a base case value of 5620.37. The blue part of the line represents the part where the uncertainty range is low and the red part represents the part where the uncertainty range is high. As a result, increasing the parameter increases the ICER value. In addition, any line crossing the threshold line means that it has a cost-effectiveness threshold. Accordingly, the base case value of the cPFNrevi parameter is 4869.18 while the base case value of the cPFNdeath parameter is 4344.34. When the parameters affecting the NMB are analysed with the hurricane diagram, the NMB value EV: 25,484.87 is represented by the dashed vertical line. This value belongs to the BHA calculated as the optimal strategy. This reveals that the largest net monetary benefit is provided by BHA (Fig. 6_ii_).

**Fig. 7:**
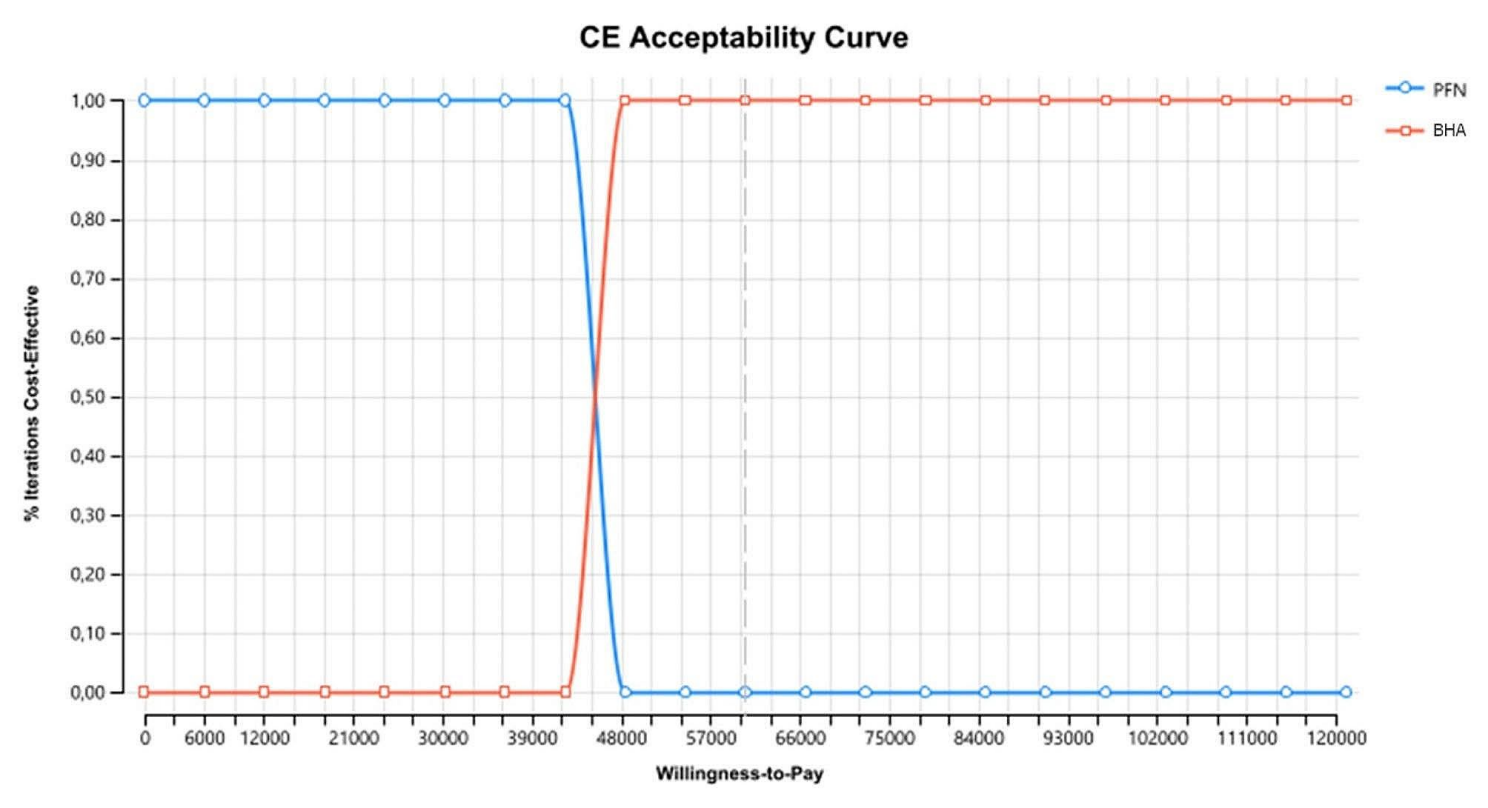
Model 1-cost effectiveness acceptability curve

For the threshold value between 0-42.367,367,50 TL, the probability of PFN being the cost-effective treatment option is found to be 1, and as the threshold value increases, the probability of BHA being the cost-effective treatment option is found to be 1. For the threshold value of 50.000 TL and above, BHA proved to be a cost-effective choice (Fig. 7).

#### Model 2

**Fig. 8:**
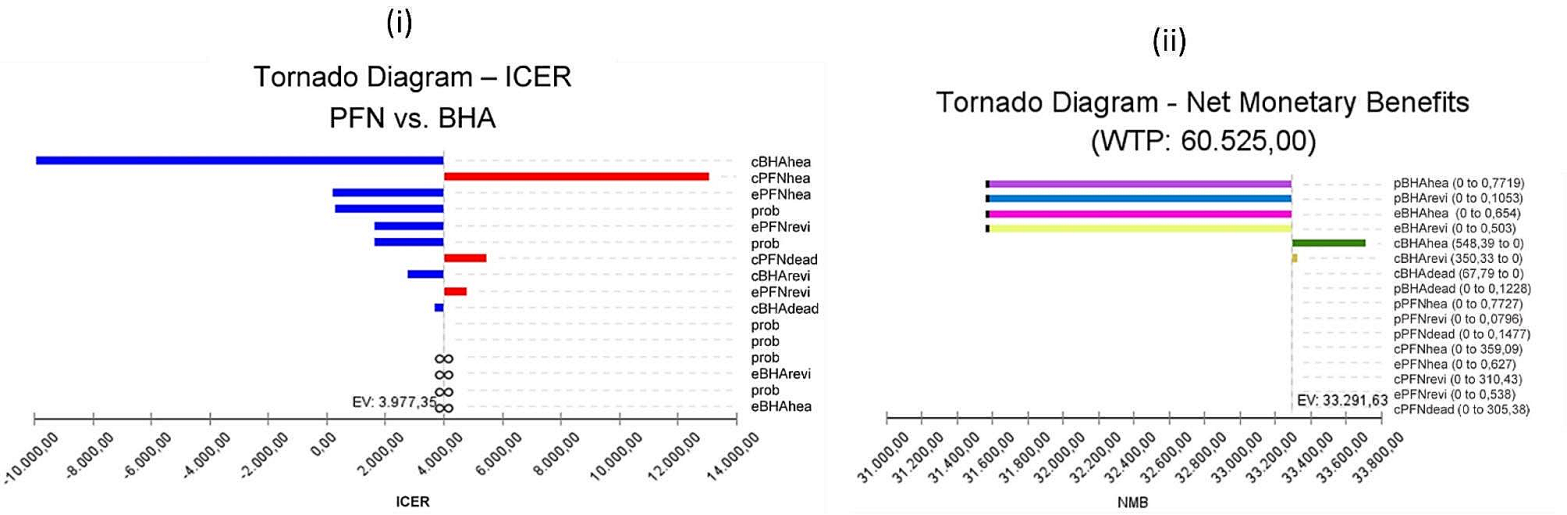
Model 2-tornado diyagram

From the patient perspective cBHAlive and cPFNlive variables can be said to have greater effect on ICER (Fig. 8_i_). The NMB value is represented by the dashed vertical line EV: 33,291.63. This value belongs to BHA, calculated to be the optimal strategy. The parameters affecting NMB were found to be BHA probability and efficacy. Additionally, the optimal strategy appears to change (Fig. 8_ii_). When the parameter values as the optimal strategy passes from PFN to BHA are investigated, it appears the cut-off point where the strategy changed was BHA procedure probability of success 0.73 and efficacy 0.62 QALY, while the revision probability was 0.05 and efficacy was 0.23 QALY.

**Fig. 9:**
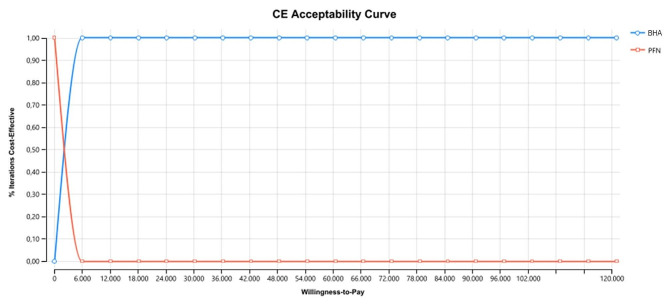
Model 2-cost effectiveness acceptability curve

With threshold value in the interval 0-6250.50 TL, the probability of PFN being the cost-effective treatment was 1, while as the threshold value increased, the probability of BHA being the cost-effective treatment was 1. In this context, for direct non-medical costs, BHA was proven to be the cost-effective choice for a threshold value of 60,525 TL (Fig. 9).

### Budget impact analysis

Within the scope of the research, the number of patients with PFN were 11,981 in 2018, 13,486 in 2019, 14,070 in 2020 and 16,259 in 2021. The number of BHA patients were 18,429 in 2018, 18,653 in 2019, 18,508 in 2020 and 18,919 in 2021. Total health expenditure planning which was 165,234 million TL in 2018, increased to 281 million TL in 2021. According to the budget impact analysis, in the period 2018–2021, treatment expenditures for PFN and BHA patients accounted for approximately 0.1% of total health expenditure. total health expenditure. In 2020, the amount spent for PFN treatment of hip fracture (43%) was 91,626,304.36 TL and the amount spent for BHA treatment (57%) was 146,362,876.51 TL. Accordingly, the share of PFN and BHA in total health expenditures was 0.4% and 0.6%, respectively.

## Discussion

In patients with hip fractures, it is necessary to balance fast and safe surgery to ensure rapid mobilization and avoid medical complications. In the literature, intramedullary nails are widely used in fracture treatment due to their biological advantages, minimally invasive approach and easy manipulation [30–32]. Internal fixation may fail in fracture treatment in elderly patients, which may lead to functional impairment. Some surgeons have turned to hip prosthesis as the primary treatment method to allow early postoperative loading, to provide rapid healing and to prevent excessive collapse of the fracture site [32–34]. Previous research has not found clear evidence of which of the alternative methods is cost-effective. In this study, a cost-effectiveness analysis was performed for the internal fixation method of the proximal femoral nail and it was determined that the bipolar hip prosthesis arthroplasty method for hip fracture was cost-effective.

In the literature, it was emphasized that the risk of fracture increases with advanced age in both men and women and that there was higher probability of hip fracture incidence in women [35, 36]. A study by Pekonen et al. (2021) found that the risk of hip fracture in women was nearly 1.7 times higher compared to men [36]. As women age, they experience more rapid bone loss compared to men and 3 out of 4 hip fractures are observed in women [37].

Falls occur before a significant portion of hip fracture [38, 39]. Research with the aim of determining the prevalence of hip fracture in the elderly and risk factors found falls (48.0%) were the primary factor (4265 participants over 65 years) [40]. Another important risk factor is previous hip fracture history in the patient. People with hip fractures have higher probability of additional fracture, including of the contralateral hip. The probability of experiencing hip fracture again among people with hip fracture is nearly two times higher [41]. The risk of recurrent hip fracture varies from 2 to 15% linked to follow-up duration [42].

In a study investigating mortality rates related to hemiarthroplasty and PFN methods, it was found that the mean hospital stay was 12.3 days. In the literature, it is stated that prolonged hospital stay has negative effects on mortality. Discharge with rehabilitation in a short time after surgery is important [43].

Zhou et al. (2019) found that the mean hospital stay of those with proximal femoral nail anti-rotation was 7.6 ± 1.8 days, while those with BHA were 6.9 ± 2.2 days [44]. In the study by Frihagen et al. (2010), the mean hospital stay was 8.2 ± 7.4 days in the internal fixation group and 10.2 ± 12 days in the hemiarthroplasty group, and no statistically significant difference was found between the two groups [19].

In our research, the ASA score for patients with PFN was 2.71, while it was 2.70 for those with BHA. Accordingly, patients had limited ability to complete daily activities and had severe systemic disease. When studies in the literature are investigated, it is notable that hip fracture patients were generally in ASA score 2 or 3. In the study by Zhou et al. (2019), the research group were found to be ASA 3 and ASA 4 [44]. In the studies conducted, it has been observed that such patient groups are ASA 2 and ASA 3 [30, 43].

As intertrochanteric femur fractures generally emerge in periods with weakened bone structure generally at advanced ages, patients have high comorbidity rates. The majority of patients in the research group had diseases like high blood pressure, diabetes, cardiovascular disease, and psychological disorders in addition to hip fracture. Accordingly, 67.60% of patients had at least one comorbid disease. A study found that the patient group with hip fracture had nearly two times more comorbidities compared to a control group. Additionally, though 17% of hip fracture patients had dementia, this rate was 7% in the control group [45]. Jolly et al. (2019) reported that 64% of patients in the hemiarthroplasty group and 60% of PFN patients had comorbid disease [32]. Geiger et al. (2007) stated that the risk of mortality increased 78% for the presence of four or more comorbidities (RR = 1.78, *p* = 0.009) [46]. The study by Fu et al. (2021) found that 62.9% of hip fracture patients (*n* = 660) had hypertension, 33.6% had coronary heart disease and 30.5% had diabetes, with 85.9% of patients having at least one comorbid disease on attendance in hospital [47].

From the payer perspective in the research, the mean cost of PFN was 6512.18 TL, while the cost of BHA was 7908.08 TL. Among the direct medical costs to hip fracture patients, the highest share comprises costs due to hospitalization. For both surgical methods, the largest share within the total costs were for materials and surgical outgoings and bed fees. From the patient perspective in the research, the cost of PFN was mean 347.28 TL, while the cost for BHA was calculated as 468.52 TL. For both surgical methods, the greatest share within the total costs were medications and medical materials. When the literature related to costs of surgical methods is investigated, hip fracture costs were high, and internal fixation was the primary treatment choice due to shorter hospitalization duration and being a lower cost method [20, 48, 49]. A randomized controlled cost study calculated costs as €47,156 for internal fixation and €38,615 for partial hip prosthesis from the social perspective with two-year follow-up of patients with internal fixation and partial hip prosthesis (*p* = 0.09). Additionally, due to the much higher risk of revision after internal fixation surgery, hemiarthroplasty was determined to have more appropriate costs in the long term [19]. Though it is very hard to compare numbers in this study with those in the international literature due to foreign exchange differences, differences in treatment protocols and cost differences, cost data from research in the literature is presented. In this context, among osteoporosis-linked fractures, hip fractures had the highest direct medical and non-medical costs. For one year after the fracture, the direct medical cost of hip fracture is $3030, while direct non-medical costs are $2352. Among direct medical costs, the highest amounts comprise costs due to admission to hospital, while among direct non-medical costs, highest amounts are for caregivers and medical materials related to the fracture [50]. A study in Saudi Arabia by Balkhi et al. (2021), the annual mean cost per person for patients without hip fractures was $975.77, while it reached $9716.26 for patients with osteoporotic fractures [51]. A study in Ontario by Alolabi et al. (2009) calculated the annual mean cost from the payer perspective was $18,100 for internal fixation and $15,843 for hemiarthroplasty [18]. A study in the USA calculated the direct medical costs to 18,233 patients treated with hemiarthroplasty were mean $34,249. For 8153 patients with revision surgery, mean cost was found to be $51,000 [12]. A study in Norway by Frihagen et al. (2010) found the total cost due to first admission to hospital was €9044 for internal fixation and €11,887 for hemiarthroplasty. The higher cost of hemiarthroplasty compared to internal fixation was found to be statistically significant. When revision surgeries are included in these costs, costs were calculated as €21,709 for internal fixation and €19,976 for hemiarthroplasty [19]. The study by Yong et al. (2020) found the cost of hemiarthroplasty ($23,467) was lower compared to screw fixation ($25,356) [52]. Esen et al. (2017) included direct medical costs in a study performed in Gazi University Faculty of Medicine Hospital. Accordingly, the first hospital admission costs in the hemiarthroplasty group (2862 TL) were lower compared to the PFNA group (3160 TL). However, when the costs of revision operations are added, there was no significant difference between the groups, with the cost of hemiarthroplasty calculated as 3268 TL [53]. A study in China by Liu et al. (2020) included direct medical and non-medical costs and found the mean total cost for internal fixation (55,676 CNY; 56,232.76 TL) was statistically significantly lower compared to hemiarthroplasty (80,297 CNY; 81,099.97 TL) [20]. Revision surgery due to failed fixation was shown to cost nearly $30,000 and was nearly twice as expensive as primary fracture surgery [54]. A study in Holland by Burgers et al. (2016) calculated mean cost during the first year of follow-up was €23,869, while it was calculated as €26,399 for the second year. For the first 10 weeks after fracture, the mean cost was found to be €15,216 (58%). The most important cost category was hospital costs, with emergency service costs €160, imaging and diagnostic methods €219, surgery €2915 and hospitalization €5732, for a total of €9026. Another important factor affecting cost was rehabilitation institutions and care services comprising 46% of total costs (€4707). Additionally, the costs for 67 patients (27%) with arthroplasty revision after internal fixation were €28,031. In this context, revision surgery exceeds the costs for primary arthroplasty [11].

Yong et al. (2020) completed a cost effectiveness analysis for screw fixation and hemiarthroplasty for a one-year time interval. Accordingly, the additional costs of hemiarthroplasty were -$1889, with additional effectiveness of 0.23. For hemiarthroplasty, the cost per QALY was $7925, while this value was calculated as $9303 for screw fixation. Accordingly, hemiarthroplasty provided better results with lower costs [52]. A study in Norway by Waaler Bjørnelv et al. (2012) calculated the cost benefit of internal fixation and hemiarthroplasty for patients with femur neck fracture. The results of the research found additional costs according to the social perspective were -€14,160 for hemiarthroplasty, with additional effectiveness 0.15 and ICER value of -€94,400. Accordingly, hemiarthroplasty had lower cost compared to the internal fixation method with higher benefit [21]. Another study by Liu et al. (2020) evaluated the cost effectiveness of internal fixation and hemiarthroplasty in elderly patients with femur neck fracture. In the hemiarthroplasty group, the additional costs were 24,621 CNY, with additional effectiveness 0.09 and ICER 273,567. Accordingly, due to being far above the threshold value for the ICER value (43,932 CNY), hemiarthroplasty was not found to be a cost effective [20]. Iorio et al. (2001) completed cost effectiveness analysis comparing internal fixation, unipolar and bipolar hemiarthroplasty and total hip arthroplasty in terms of complication rates, mortality, reoperation rates and function. They found arthroplasty was the cost-effective method for fractures in the elderly [48].

In study of patients with 61 undergoing internal fixation and 47 hemiarthroplasties, Alolabi et al. (2009) calculated the additional costs for internal fixation as $2257 with ICER value of $64,714,103 [18]. Thorsness et al. (2018) completed a cost effectiveness analysis comparing open reduction internal fixation with hemiarthroplasty. The additional cost of open reduction internal fixation was $436, with additional effectiveness 0.08 and ICER value of $5319/QALY [55]. Slover et al. (2009) assessed the cost effectiveness over a 20-year period for total hip arthroplasty and hemiarthroplasty in 2009. As a result of the research, the total hip prosthesis additional cost was $3000, with additional effectiveness 1.53. The total hip prosthesis treatment strategy was calculated to ICER value of $1960 per QALY. Accordingly, though total hip prosthesis was more costly, due to high benefits in a select population, it was stated to be a cost-effective treatment method [12]. Another study by Burgers et al. (2016) found the additional costs of arthroplasty were €14,844 (first year) and €2539 (later year) [11] A study by Blythe et al. (2020) completed cost effectiveness analysis for cemented and uncemented hemiarthroplasty and total hip arthroplasty. The research results showed that uncemented hemiarthroplasty was more costly compared to cemented hemiarthroplasty and total hip arthroplasty and caused worse health outcomes. The transition to cemented arthroplasty was stated to provide 2 million dollars of savings and 203 QALY gains over 5 years to the Australian health system [56]. A study by Larranaga et al. (2021) performed cost benefit analysis of total and partial hip prosthesis. Accordingly, total hip prosthesis had higher cost and higher benefit (€2465 and 0.42 QALY) [10].

Some policy recommendations can be made based on the research findings. Most patients included within the scope of the research broke their hips due to falls. As the majority of falls are preventable, it is important to increase access to information and awareness about preventing falls in the high-risk group, risks should be determined in the elderly and a variety of interventions encouraged, risk factors should be reduced and integrated care should be provided for elderly patients who fall. Additionally, in the transition period after discharge for elderly people with hip fracture, they are very dependent on caregivers due to limited ability to complete activities of daily living. During discharge, patients and caregivers may be given education about hip fracture injury, healing plans and care responsibilities and a written brochure may be designed. Additionally, as most patients do not have elevators in the home, returning from hospital/clinics may be difficult. Health service providers and policy makers should assess costs along with quality of life and satisfaction. In this way, the economic assessment of interventions will be more realistic and service quality may be regularly monitored. Within the scope of cost effectiveness studies, it is recommended that all countries prepare cost effectiveness guides, as in Belgium, England, Italy and Holland. Finally, there are some limitations to this study. Firstly, interviews after operations were completed by phone due to the COVID-19 pandemic, which may have limited understandability. Secondly, XXBLINDEDXX operate in the research hospital. It is assumed there were no differences in processes and procedures related to the surgery. As there were no specific scores for Türkiye in analyses related to EQ-5D-5 L quality of life, scores for Germany were used. Finally, this study only includes individuals over 65 years of age with a fractured femoral neck.

Though our analysis shows that BHA is the choice with most appropriate cost to treat hip fractures, data are not sufficiently robust or detailed to propose that it is the superior choice for all patients or conditions. Instead, our research findings offer an additional lens through which surgeons can examine costs and outcomes at the point of making decisions.

## Conclusion

It is important to determine the cost-effective method due to the increasing incidence of hip fracture and burden on the economy. Especially for patients with PFN surgery, revision costs were found to be higher. The transition from internal fixation to arthroplasty is even more costly than primary arthroplasty, emphasizing the need to carefully select the primary treatment for hip fractures. Cost should most definitely not be the primary factor affecting selection of surgical procedures, while it is an element that should be noted for fracture treatment in the current medical environment with increasing costs and limited resources. This study proposes that higher cost should not be an obstacle to BHA. The cost effectiveness acceptability curve provides information about the uncertainty related to the cost effectiveness of interventions. In spite of initial costs, BHA results in higher quality of life and was shown to be more cost effective compared to PFN. The basic aim of cost effectiveness analysis is to guide clinicians and paying institutions by offering different choices, rather than being based on selection of a superior alternative. The preliminary results obtained in this research contribute to this field of science and provide beneficial information for payer institutions.

## Data Availability

No datasets were generated or analysed during the current study.
